# Outlining the global variation in resources for traumatic brain injury care: site-level data from the Global Neurotrauma Outcomes Study (GNOS)

**DOI:** 10.1136/bmjgh-2025-023154

**Published:** 2026-04-28

**Authors:** Adarsh Arun Menon, David Clark, Michael F Bath, Sara Venturini, Mohammed Bashir, Alexis Joannides, Amos O Adeleye, Abdul Hafid Bajamal, Hagos Biluts, Karol Budohoski, Ari Ercole, Rocío Fernández-Méndez, Anthony Figaji, Deepak Gupta, Roger Härtl, Corrado Iaccarino, Tariq Khan, Tsegazeab Laeke, Andres M Rubiano, Hamisi K Shabani, Kachinga Sichizya, Manoj Tewari, Abenezer Tirsit, Myat Thu, Manjul Tripathi, Rikin Trivedi, Bhagavatula Indira Devi, Franco Servadei, David Menon, Angelos Kolias, Tom Bashford, Peter Hutchinson, Global Neurotrauma Outcomes Study collaborative

**Affiliations:** 1 International Health Systems Group, University of Cambridge Department of Engineering, Cambridge, UK; 2 NIHR Global Health Research Group on Neurotrauma, Cambridge, England, UK; 3 International Health Systems Group, Department of Engineering, University of Cambridge, Cambridge, UK; 4 Department of Surgery, University of Ibadan College of Medicine, Ibadan, Oyo, Nigeria; 5 Department of Neurosurgery, Dr Soetomo Regional General Hospital, Surabaya, East Java, Indonesia; 6 Neurosurgery Unit, Department of Surgery, Addis Ababa University College of Health Sciences, Addis Ababa, Addis Ababa, Ethiopia; 7 Division of Neurosurgery, Red Cross Children’s Hospital & University of Cape Town, Cape Town, South Africa; 8 Department of Neurosurgery, All India Institute of Ayurveda, New Delhi, DL, India; 9 Department of Neurological Surgery, Weill Cornell Medicine, New York, New York, USA; 10 Neurosurgery Division, University Hospital of Parma, Parma, Emilia-Romagna, Italy; 11 Northwest General Hospital & Research Centre, Peshawar, N.W.F.P, Pakistan; 12 Neurosurgery Unit, Addis Ababa University College of Health Sciences, Addis Ababa, Addis Ababa, Ethiopia; 13 Neurosciences Institute, Universidad El Bosque, Bogota, Cundinamarca, Colombia; 14 Muhimbili Orthopedic and Neurosurgery Institute, Dar es Salaam, Tanzania; 15 Neurosurgery Division, University of Zambia University Teaching Hospital, Lusaka, Lusaka Province, Zambia; 16 Post Graduate Institute of Medical Education and Research, Chandigarh, CH, India; 17 Department of Neurosurgery, Yangon General Hospital, Yangon, Yangon Region, Myanmar; 18 Department of Neurosurgery, Post Graduate Institute of Medical Education and Research, Chandigarh, CH, India; 19 Department of Neurosurgery, National Institute of Mental Health and Neuro Sciences, Bangalore, India; 20 Fondazione IRCCS Istituto Neurologico Carlo Besta, Milan, Italy; 21 Division of Anaesthesia, Department of Medicine, University of Cambridge, Cambridge, England, UK

**Keywords:** Global Health, Neurology, Traumatology, Health systems

## Abstract

**Introduction:**

Traumatic brain injury (TBI), a leading cause of global morbidity and mortality, has notably poorer outcomes in resource-poor settings. Managing TBI effectively requires functional integration of several interdependent phases of care which, in resource-poor settings, may be both individually fragile and poorly coordinated. However, there is a paucity of global data quantifying systemic deficiencies in TBI care across varying resource settings which hinders targeted improvement. To address this, we present an analysis of site surveys from centres contributing to the Global Neurotrauma Outcomes Study, to illustrate the global variation in health system performance with respect to TBI management.

**Methods:**

The GNOS was a prospective, international, multicentre cohort study conducted between 1 November 2018 and 31 January 2020 across 159 centres that perform emergency surgery for TBI. Participating centres completed a 50-point site survey, assessing local resource availability and guideline usage across the TBI care pathway, including prehospital, intraoperative and rehabilitative care. Responses were stratified by each country’s Human Development Index (HDI).

**Results:**

The site survey was completed by 153/159 (96%) centres. Variation in resource availability across HDI strata was least prevalent within operating theatres, whereas the biggest disparities were observed in prehospital and rehabilitation care. Resource-poor settings reported deficiencies in both the capital resources and skilled human resources needed to deliver TBI care. Overall, participants most commonly suggested that improvements in prehospital care would have the greatest impact on patient outcomes in TBI (53/153, 35%), although participants from the more resource-poor settings more commonly suggested improvements in intensive care management (6/14, 43%).

**Conclusion:**

There are profound HDI-associated disparities in the resources for TBI care, which likely account for the global variation in patient mortality. Our study suggests improving the provision of non-operative interventions within TBI care pathways may offer the most successful approach to improve patient outcomes.

**Trial registration number:**

NCT04212754.

**WHAT IS ALREADY KNOWN ON THIS TOPIC**

- No previously published studies have probed in detail the resource availability within traumatic brain injury (TBI) care pathways globally; there is a particular lack of data for low- and middle-income countries.

**WHAT THIS STUDY ADDS**

- The data on resource availability presented here are essential to describe the landscape of global TBI care.
- It demonstrates that across the entire spectrum of human development index (HDI) settings, intraoperative care for TBI was perceived as the most well-resourced, while global resource disparities appeared most pronounced in the prehospital and rehabilitation phases of care.

**HOW THIS STUDY MIGHT AFFECT RESEARCH, PRACTICE OR POLICY**

- Having identified areas of relative resource deficiencies for TBI care globally, our study offers valuable considerations for worldwide policy on the strengthening of TBI care pathways within health systems.

## Introduction

 Traumatic brain injury (TBI) is a major global public health concern, believed to affect as many as 69 million people annually^1^ and accounting for approximately one-third of all injury-related deaths.^2^ TBI is also a significant driver of morbidity, with the 2016 Global Burden of Disease Study estimating that it resulted in 8.1 million years lived with disability.^3^ The strain imposed by TBI on health systems is particularly pronounced in low- and middle-income countries (LMICs), where the total annual incidence of TBI cases requiring neurosurgical intervention is estimated at 4.5 million.^4^ Meeting this demand is inevitably difficult, especially considering the fragility of LMIC healthcare systems,^5 6^ and the highly technical, resource-intensive, multidisciplinary and time-critical processes necessary for safe and effective TBI care.^7^,^10^ However, this challenge is universal, including in high-income countries, as the scarcity and geographic dilution of resources in rural areas manifest in a higher TBI mortality rate.^11^

In 2022, the Global Neurotrauma Outcomes Study (GNOS) reported a prospective global cohort study that examined the worldwide management and outcomes of patients undergoing emergency surgery for TBI.^12^ It demonstrated significant associations between the national Human Development Index (HDI) and casemix, mechanism of injury, demographic variation and mortality. This provided an epidemiological picture of TBI in LMICs being predominantly a result of road traffic injuries in young male patients who suffer a greater degree of mortality than in high-income settings, consistent with existing evidence from smaller studies and the clinical experience of LMIC practitioners.^13^ GNOS focused on TBI requiring inpatient neurosurgical care, on the basis that the overwhelming burden of mortality and the vast majority of late functional disability comes from this section of TBI.

However, epidemiological data are limited in their ability to describe the global variability in the structures and processes required to deliver TBI care, and understanding these is crucial to identifying future health system interventions. While the three-delays model, derived from obstetrics, has previously been used to understand TBI care pathways and identify potential foci for improvement, TBI management is complex and this reduction could be seen as an oversimplification of a challenging area.^5 14 15^ As an example, while the management of severe TBI requires maintenance of adequate cerebral perfusion and avoidance of hypoxia, pragmatically this relies on the functional ability of the healthcare system to deliver sedation, intubation, mechanical ventilation, vasopressor support and invasive haemodynamic monitoring across prehospital, acute and intensive care domains, before considering neurosurgical approaches for managing raised intracranial pressure.^16^

To provide a global perspective on the care pathways for TBI across income settings, the GNOS study employed a detailed site-level survey across participating centres. The survey aimed to assess the availability of resources across each phase of the TBI care pathway (prehospital, emergency department, operating theatres, recovery/intensive care unit (ICU) and rehabilitation services) as well as the subjective opinion of participating centres surrounding which of these phases of care they deemed most in need of improvement. We report the results of this site survey, which provides an insight into the current landscape of global TBI care, aiming to both understand some of the causal variables for the global disparities observed in TBI care and offer suggestions as to the most important considerations required to improve global TBI care.

## Methods

GNOS was a prospective, international, multicentre cohort study conducted between 1 November 2018 and 31 Jan 2020, open to any centre worldwide that performs emergency surgery for TBI. The full study protocol and its primary epidemiological findings have previously been described.^12 17^

### Study population

A total of 159 centres across 57 countries were recruited and submitted data for TBI admissions requiring emergency surgery. Centres could select any continuous 30-day interval during the study period in which to collect data for GNOS. Countries were stratified based on their HDI score, a composite measure devised by the United Nations Development Programme that incorporates health, education and living standards to quantify the level of development of a given country.^18^ HDI values range from 0.000 to 1.000 and are categorised as either very high (VH-HDI, ≥0.800), high (H-HDI, 0.700–0.799), medium (M-HDI, 0.550–0.799) or low (L-HDI, <0.550).^18^

### Development of the site survey

Prior to data collection, a survey was constructed to characterise TBI management at each centre. This was designed through an iterative process by a multidisciplinary protocol development group, comprising clinicians caring for TBI patients from a variety of human development settings. An initial survey was piloted in Zambia and, after edits were incorporated, a final version was generated and approved by the World Federation of Neurosurgeons’ neurotrauma committee.

The resultant qualitative-descriptive survey included 50 items, assessing resource availability and guideline usage at each phase of the TBI care pathway, including prehospital, acute and follow-up/rehabilitative care (online supplemental Appendix A). Local research teams completing the site surveys were also asked to indicate, in their opinion, at which phase of TBI management improvements would have the biggest local impact on patient care.

### Distribution of the site survey

The survey, along with the case report form used in the primary analysis of GNOS, was distributed digitally, using the Qualtrics platform (https://cambridge.eu.qualtrics.com), and was completed by the study team at each centre. In cases where multiple teams in each centre participated, each team was required to answer the survey independently, allowing assessment of inter-rater reliability of the survey items.

The ORION cloud-based platform (Obex Technologies) was used for the collection of patient-level data. ORION is a combined research and clinical data network, which was established within the University of Cambridge in 2012 (https://orioncloud.org/). It hosts registries, research databases and clinical service tools across multiple institutions and acted as both the collection tool and repository for all GNOS data.

### Statistical analysis

Descriptive statistics are reported as counts and percentages. Proportions were compared between centres of different HDI strata and p values were derived using χ² tests. Statistical significance was two-sided and fixed at p<0.05. All analyses were conducted using R version 4.1.1.

### Study registration

GNOS was registered on ClinicalTrials.gov (NCT04212754) and the Clinical Trials Registry—India (CTRI/2019/02/017479). The study was funded and conducted by the National Institute for Health Research Global Health Research Group on Neurotrauma (NIHR16/137/105). A UK National Health Service Research Ethics Service considered this study exempt from formal research registration (Southeast Scotland Research Ethics Service) but, before commencing data entry, each contributing team was required to submit evidence of appropriate local approval of the study.

### Patient and public involvement statement

Findings from the initial publication that arose from the GNOS have been actively disseminated to the general public via both social media and internal media networks of the University of Cambridge and Cambridge University Hospitals NHS Foundation Trust,^19^,^21^ with active plans to similarly share findings from this work.

### Funding statement

Funding was received from the National Institute for Health Research Global Health Research Group. The funder of the study had no role in study design, data collection, data analysis, data interpretation or writing of the report.

## Results

### Demographics

**Figure 1:**
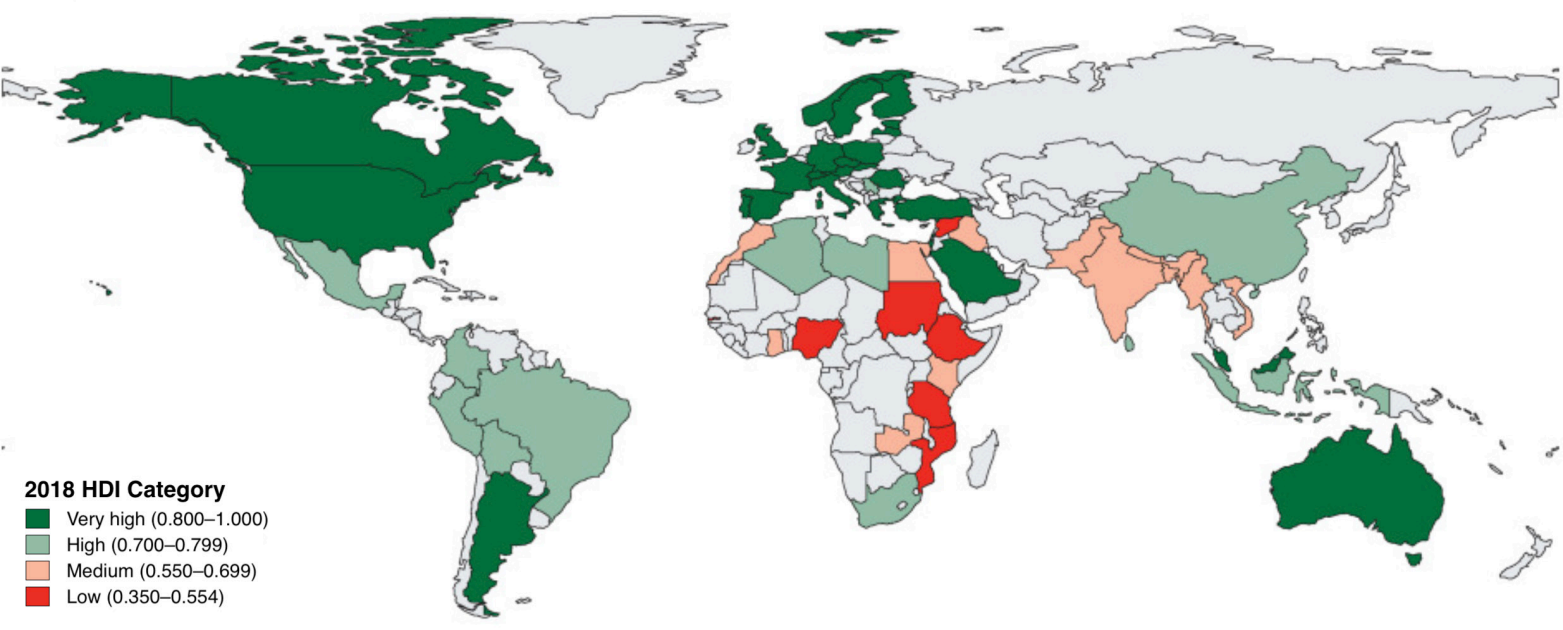
Global distribution of GNOS centres. Countries with centres that participated in the GNOS site-level survey are shaded according to the HDI category of the country. Adapted with permission from Clark *et al.*^12^ GNOS, Global Neurotrauma Outcomes Study; HDI, Human Development Index.

The site survey was completed and returned by 153/159 (96%) centres. Participating centres were spread across VH-HDI (51%), H-HDI (22%), M-HDI (18%) and L-HDI (9%) countries (figure 1). Hospitals were primarily government funded (85%) and the vast majority were in urban areas (96%). All centres had an area identified as an ICU—defined as a unit with at least one mechanical ventilator and where patients are expected to be admitted for at least 24 hours.

### Phases of care for improvement

Across all centres, the prehospital phase was identified as being most in need of improvement, with the surgical phase of TBI care the lowest priority for intervention (table 1). However, there was notable inter-HDI variability in the modal choice for the area most in need of development: for VH-HDI centres, this was rehabilitation; H-HDI and M-HDI centres chose prehospital care and L-HDI centres intimated that improvements in the intensive care management of their TBI patients would be most valuable.

**Table 1:** Site-level survey data on phases of TBI care and availability of guidelines in each phase

	VH-HDI (n=78)	H-HDI (n=33)	M-HDI (n=28)	L-HDI (n=14)	Total (n=153)	P value
**If you had to pick a single phase of TBI management in your institution to improve, which one do you think would have the greatest impact on patient outcome?**						0.043
Prehospital care	19 (24%)	15 (45%)	14 (50%)	5 (36%)	53 (35%)	
Initial management in the emergency department	14 (19%)	5 (15%)	4 (14%)	3 (21%)	26 (17%)	
Surgery	1 (1%)	1 (3%)	1 (4%)	0 (0%)	3 (2%)	
Intensive care management	17 (22%)	9 (27%)	5 (18%)	6 (43%)	37 (24%)	
Rehabilitation	27 (35%)	3 (9%)	4 (14%)	0 (0%)	34 (22%)	
**Do you use any guidelines to assist with the management of TBI?**	73 (94%)	30 (91%)	26 (93%)	9 (64%)	138 (90%)	0.008
**Phases of care for which there are guidelines in your institution**						
Prehospital care	30 (39%)	5 (15%)	6 (21%)	0 (0%)	41 (27%)	
Emergency department	47 (60%)	20 (61%)	19 (68%)	7 (50%)	93 (61%)	
Selecting patients for CT head	49 (63%)	15 (45%)	22 (79%)	8 (57%)	41 (61%)	
Intensive care management	57 (73%)	20 (61%)	21 (75%)	6 (43%)	104 (68%)	
Treatment of raised ICP	61 (78%)	14 (42%)	22 (79%)	5 (36%)	102 (67%)	
Rehabilitation	15 (19%)	4 (12%)	8 (29%)	1 (7%)	28 (18%)	

Data are stratified by HDI tier of the countries in which each participating centre was situated.

Data are presented as n (%), and p values are derived from χ² analyses.

HDI, Human Development Index; H-HDI, high HDI; ICP, intracranial pressure; L-HDI, low HDI; M-HDI, medium HDI; TBI, traumatic brain injury; VH-HDI, very high HDI.

### Protocolisation of care

Guideline utilisation was >90% in all but the L-HDI tier (table 1). Guidelines were most used in intensive care and in the management of raised ICP and least used in prehospital care and rehabilitation. Disparities in guidelines usage between HDI tiers were seen throughout but were most marked during the prehospital and rehabilitation phases. No L-HDI centre used guidelines in prehospital care for TBI.

### Resource availability

Resources differed significantly between HDI tiers at all phases and were more abundant in higher HDI centres. L-HDI centres had resource deficiencies in all phases, but these were most pronounced in the prehospital, ICU and rehabilitation phases of management. M-HDI centres similarly had deficiencies in the prehospital and rehabilitation phases. H-HDI centres had deficiencies during the rehabilitation phase but good resource availability elsewhere, and VH-HDI centres had excellent resource availability with some deficiencies in the rehabilitation phase.

#### Initial management: prehospital and emergency department

The prehospital phase included availability of two items, pulse oximetry and supplemental oxygen, both of which decreased significantly from the higher to lower HDI strata, with no L-HDI centre having regular access to supplemental oxygen. The emergency care phase assessed the above two items, but also included trauma teams, the latter of which were present in most centres across all HDI strata. Pulse oximeters and supplemental oxygen were more readily available than in the prehospital phase across all HDI strata (table 2).

**Table 2:** Resource availability within the initial phases of TBI care

	VH-HDI (n=78)	H-HDI (n=33)	M-HDI (n=28)	L-HDI (n=14)	Total (n=153)	P value
**Do you have a trauma team who immediately assesses seriously injured patients when they first arrive at your trauma institution?**						<0.001
All of the time	65 (83%)	17 (52%)	17 (61%)	4 (29%)	103 (67%)	
Most of the time	4 (5%)	9 (27%)	9 (32%)	7 (50%)	29 (19%)	
Some of the time	1 (1%)	3 (9%)	1 (4%)	2 (14%)	7 (5%)	
None of the time	8 (10%)	4 (12%)	1 (4%)	1 (7%)	14 (9%)	
**How often is a pulse oximeter available in the following locations in your institution?**						
Prehospital						<0.001
All of the time	69 (88%)	12 (36%)	3 (11%)	0 (0%)	84 (55%)	
Most of the time	8 (10%)	8 (24%)	5 (18%)	0 (0%)	21 (14%)	
Some of the time	1 (1%)	13 (39%)	13 (46%)	5 (36%)	32 (21%)	
None of the time	0 (0%)	0 (0%)	7 (25%)	9 (64%)	16 (10%)	
Emergency department						<0.001
All of the time	78 (100%)	26 (79%)	19 (68%)	6 (43%)	129 (84%)	
Most of the time	0 (0%)	7 (21%)	7 (25%)	4 (29%)	18 (12%)	
Some of the time	0 (0%)	0 (0%)	2 (7%)	4 (29%)	6 (4%)	
None of the time	0 (0%)	0 (0%)	0 (0%)	0 (0%)	0 (0%)	
**How often is supplemental oxygen available in the following settings at your institution?**						
Prehospital						<0.001
All of the time	69 (88%)	14 (42%)	7 (25%)	0 (0%)	90 (59%)	
Most of the time	8 (10%)	13 (39%)	7 (25%)	1 (7%)	29 (19%)	
Some of the time	1 (1%)	6 (18%)	11 (39%)	6 (43%)	24 (16%)	
None of the time	0 (0%)	0 (0%)	3 (11%)	7 (50%)	10 (7%)	
Emergency department						<0.001
All of the time	77 (99%)	33 (100%)	26 (93%)	8 (57%)	144 (94%)	
Most of the time	1 (1%)	0 (0%)	1 (4%)	5 (36%)	7 (5%)	
Some of the time	0 (0%)	0 (0%)	1 (4%)	1 (7%)	2 (1%)	
None of the time	0 (0%)	0 (0%)	0 (0%)	0 (0%)	0 (0%)	
Neurosurgical ward(s)						<0.001
All of the time	71 (91%)	22 (67%)	22 (79%)	5 (36%)	120 (78%)	
Most of the time	6 (8%)	5 (15%)	5 (18%)	6 (43%)	22 (14%)	
Some of the time	1 (1%)	5 (15%)	1 (4%)	2 (14%)	9 (6%)	
None of the time	0 (0%)	1 (3%)	0 (0%)	1 (7%)	2 (1%)	

Availability of supplemental oxygen on neurosurgical wards is also presented here. Data are stratified by HDI tier of the countries in which each participating centre was situated. Data are presented as n (%), and p values are derived from χ² analyses.

HDI, Human Development Index; H-HDI, high HDI; L-HDI, low HDI; M-HDI, medium HDI; TBI, traumatic brain injury; VH-HDI, very high HDI.

#### Operating theatre and post-operative resources

Pulse oximetry and supplemental oxygen were always available in operating theatres, except in two L-HDI centres (online supplemental Appendix B). Only in one M-HDI and one L-HDI centre was pulse oximetry not available at least most of the time in recovery (post-anaesthesia care) units. In contrast, there was a notable HDI-dependent decrease in supplemental oxygen availability in ward-based settings (table 2).

Surgical equipment was available in most centres regardless of HDI tier, however, the availability of intraoperative haemostatic agents and bone wax was more limited in L-HDI centres (table 3).

**Table 3:** Availability of key neurosurgical and neurointensive care resources

	VH-HDI (N=78)	H-HDI (N=33)	M-HDI (N=28)	L-HDI (N=14)	Total (N=153)	P value
**SURGERY**						
**Access to neurosurgical equipment**						
Bone wax						<0.001
All of the time	74 (95%)	24 (73%)	23 (82%)	3 (21%)	124 (81%)	
Most of the time	4 (5%)	6 (18%)	4 (14%)	6 (43%)	20 (13%)	
Some of the time	0 (0%)	3 (9%)	1 (4%)	4 (29%)	8 (5%)	
Never	0 (0%)	0 (0%)	0 (0%)	1 (7%)	1 (1%)	
Intraoperative haemostatic agents						<0.001
All of the time	73 (94%)	20 (61%)	25 (89%)	4 (29%)	122 (80%)	
Most of the time	4 (5%)	10 (30%)	3 (11%)	5 (36%)	22 (14%)	
Some of the time	1 (1%)	2 (6%)	0 (0%)	3 (21%)	6 (4%)	
Never	0 (0%)	1 (3%)	0 (0%)	2 (14%)	3 (2%)	
**INTENSIVE CARE**						
**How would you best describe the ICU in your hospital that TBI patients are typically admitted to?**						<0.001
General ICU—there is only one ICU in our hospital	27 (35%)	22 (67%)	5 (18%)	9 (64%)	63 (41%)	
Medical ICU	0 (0%)	2 (6%)	0 (0%)	0 (0%)	2 (1%)	
Neuro ICU	37 (47%)	6 (18%)	17 (61%)	1 (7%)	61 (40%)	
Surgical ICU	11 (14%)	0 (0%)	5 (18%)	4 (29%)	20 (13%)	
Trauma ICU	3 (4%)	3 (9%)	1 (4%)	0 (0%)	7 (5%)	
**Managing severe TBI (GCS 3–8)**						
Availability of mechanical ventilator for severe TBI when needed						<0.001
All of the time	74 (95%)	24 (73%)	12 (43%)	2 (14%)	112 (73%)	
Most of the time	3 (4%)	9 (27%)	10 (36%)	7 (50%)	29 (19%)	
Some of the time	1 (1%)	0 (0%)	6 (21%)	4 (29%)	11 (7%)	
None of the time	0 (0%)	0 (0%)	0 (0%)	1 (7%)	1 (1%)	
Invasive blood pressure (via arterial line)						<0.001
All of the time	75 (96%)	18 (55%)	11 (39%)	0 (0%)	104 (68%)	
Most of the time	3 (4%)	4 (12%)	2 (7%)	1 (7%)	10 (7%)	
Some of the time	0 (0%)	9 (27%)	10 (36%)	4 (29%)	23 (15%)	
None of the time	0 (0%)	2 (6%)	5 (18%)	9 (64%)	16 (10%)	
Arterial blood gas testing						<0.001
All of the time	76 (97%)	29 (88%)	19 (68%)	3 (21%)	127 (83%)	
Most of the time	2 (3%)	2 (6%)	5 (18%)	1 (7%)	10 (7%)	
Some of the time	0 (0%)	2 (6%)	2 (7%)	2 (14%)	6 (4%)	
None of the time	0 (0%)	0 (0%)	2 (7%)	8 (57%)	10 (7%)	
Waveform capnography						<0.001
All of the time	73 (94%)	10 (30%)	10 (36%)	1 (7%)	94 (61%)	
Most of the time	2 (3%)	13 (39%)	7 (25%)	0 (0%)	22 (14%)	
Some of the time	2 (3%)	6 (18%)	9 (32%)	4 (29%)	21 (14%)	
None of the time	1 (1%)	4 (12%)	2 (7%)	9 (64%)	16 (10%)	
Serum electrolytes testing						<0.001
All of the time	78 (100%)	31 (94%)	27 (96%)	5 (36%)	141 (92%)	
Most of the time	0 (0%)	2 (6%)	1 (4%)	6 (43%)	9 (6%)	
Some of the time	0 (0%)	0 (0%)	0 (0.0%)	3 (21%)	3 (2%)	
None of the time	0 (0%)	0 (0%)	0 (0.0%)	0 (0%)	0 (0%)	
Sedatives						<0.001
All of the time	77 (99%)	31 (94%)	27 (96%)	8 (57%)	143 (94%)	
Most of the time	1 (1%)	2 (6%)	1 (4%)	4 (29%)	8 (5%)	
Some of the time	0 (0%)	0 (0%)	0 (0%)	2 (14%)	2 (1%)	
None of the time	0 (0%)	0 (0%)	0 (0%)	0 (0%)	0 (0%)	
Muscle relaxants						<0.001
All of the time	76 (97%)	30 (91%)	25 (89%)	7 (50%)	138 (90%)	
Most of the time	1 (1%)	2 (6%)	0 (0%)	4 (29%)	7 (5%)	
Some of the time	1 (1%)	1 (3%)	2 (7%)	2 (14%)	6 (4%)	
None of the time	0 (0%)	0 (0%)	1 (4%)	1 (7%)	2 (1%)	
Hyperosmolar therapy (e.g. mannitol or hypertonic saline)						<0.001
All of the time	77 (99%)	31 (94%)	26 (93%)	7 (50%)	141 (92%)	
Most of the time	1 (1%)	2 (6%)	0 (0%)	6 (43%)	9 (6%)	
Some of the time	0 (0%)	0 (0%)	2 (7%)	1 (7%)	3 (2%)	
None of the time	0 (0%)	0 (0%)	0 (0%)	0 (0%)	0 (0%)	
Erythrocyte transfusion						<0.001
All of the time	76 (97%)	27 (82%)	22 (79%)	6 (43%)	131 (86%)	
Most of the time	1 (1%)	4 (12%)	4 (14%)	4 (29%)	13 (8%)	
Some of the time	1 (1%)	2 (6%)	2 (7%)	1 (7%)	6 (4%)	
None of the time	0 (0%)	0 (0%)	0 (0%)	3 (21%)	3 (2%)	
Availability of enteral/parenteral nutrition to maintain nutritional requirements for severe TBI patients when needed						0.002
All of the time	73 (94%)	28 (85%)	24 (86%)	7 (50%)	132 (86%)	
Most of the time	5 (6%)	4 (12%)	3 (11%)	5 (36%)	17 (11%)	
Some of the time	0 (0%)	1 (3%)	1 (4%)	2 (14%)	4 (3%)	
None of the time	0 (0%)	0 (0%)	0 (0%)	0 (0%)	0 (0%)	

Data are stratified by HDI tier of the countries in which each participating centre was situated. Data are presented as n (%), and p values are derived from χ² analyses.

GCS, Glasgow Coma Scale; HDI, Human Development Index; ICU, intensive care unit; TBI, traumatic brain injury.

All the requisite intensive care resources were readily available in VH-HDI centres and were less available in centres of lower HDI (table 3). The largest disparities were seen in capnography along with invasive arterial and central venous pressure monitoring (online supplemental Appendix B), with less than 20% of L-HDI centres reporting regular access to these modalities.

#### Follow-up after severe TBI

Of 153 centres, 71 centres (46%) followed up ≥75% of severe TBI patients in an outpatient clinic and 49 centres (32%) followed up <50% of such patients. The proportion of severe TBI patients receiving clinic follow-up diminished with decreasing HDI (online supplemental Appendix B); the ≥75% follow-up rate was met by 45/78 (58%) VH-HDI centres, but only 3/14 (21%) L-HDI centres.

Among rehabilitation staff, physiotherapists were most prevalent and were regularly available in most centres except for L-HDI centres, of which 71% indicated regular access. Other staff such as neuropsychologists, speech and language therapists, rehabilitation medicine physicians and occupational therapists were less commonly available, and their availability decreased significantly in lower HDI strata (table 4).

**Table 4:** Access to rehabilitation professionals post-TBI

	VH-HDI (n=78)	H-HDI (n=33)	M-HDI (n=28)	L-HDI (n=14)	Total (n=153)	P value
**Access to healthcare professionals for severe TBI patients after the acute period of their illness**						
Physiotherapist						0.006
All patients	65 (83%)	18 (55%)	20 (71%)	5 (36%)	108 (71%)	
Most patients	10 (13%)	8 (24%)	5 (18%)	5 (36%)	28 (18%)	
Some patients	2 (3%)	7 (21%)	3 (11%)	4 (29%)	16 (10%)	
No patients	1 (1%)	0 (0%)	0 (0%)	0 (0%)	1 (1%)	
Occupational therapist						<0.001
All patients	40 (51%)	7 (21%)	3 (11%)	0 (0%)	50 (33%)	
Most patients	12 (15%)	7 (21%)	6 (21%)	1 (7%)	26 (17%)	
Some patients	13 (17%)	8 (24%)	8 (29%)	6 (43%)	35 (23%)	
No patients	13 (17%)	11 (33%)	11 (39%)	7 (50%)	42 (27%)	
Neuropsychologist						<0.001
All patients	24 (31%)	2 (6%)	3 (11%)	0 (0%)	29 (19%)	
Most patients	15 (19%)	2 (6%)	2 (7%)	1 (7%)	20 (13%)	
Some patients	28 (36%)	14 (42%)	9 (32%)	4 (29%)	55 (36%)	
No patients	11 (14%)	15 (46%)	14 (50%)	9 (64%)	49 (32%)	
Speech and language therapist						<0.001
All patients	41 (53%)	7 (21%)	3 (11%)	0 (0%)	51 (33%)	
Most patients	12 (15%)	6 (18%)	8 (29%)	0 (0%)	26 (17%)	
Some patients	18 (23%)	11 (33%)	8 (29%)	6 (43%)	43 (28%)	
No patients	7 (9%)	9 (27%)	9 (32%)	8 (57%)	33 (22%)	
Rehabilitation medicine physician						<0.001
All patients	47 (60%)	8 (24%)	5 (18%)	1 (7%)	61 (40%)	
Most patients	17 (22%)	4 (12%)	4 (14%)	3 (21%)	28 (18%)	
Some patients	8 (10%)	10 (30%)	7 (25%)	1 (7%)	26 (17%)	
No patients	6 (8%)	11 (33%)	12 (43%)	9 (64%)	38 (25%)	

Data are stratified by HDI tier of the countries in which each participating centre was situated. Data are presented as n (%), and p values are derived from χ² analyses.

HDI, Human Development Index; H-HDI, high HDI; L-HDI, low HDI; M-HDI, medium HDI; TBI, traumatic brain injury; VH-HDI, very high HDI.

## Discussion

We present the results of a global survey characterising the provision of care across different phases in 153 centres offering emergency neurosurgery for the management of TBI across a range of human development settings. TBI is a complex and heterogeneous clinical entity, with notable epidemiological variation across the HDI spectrum.^8^ Our data demonstrate marked HDI-associated disparities in the resources available for TBI care, most pronounced within the prehospital and rehabilitation phases of care. Centres in (V)H-HDI settings reported greater access to specialist clinical roles (ranging from trauma teams to rehabilitation specialists) and equipment and tended to suggest that improving rehabilitation care would be most impactful. Meanwhile, centres in lower HDI contexts most frequently identified prehospital improvements as the highest priority for impact. This underscores the need for context-specific strategies to improve TBI outcomes globally.

Traditionally, it has been difficult to gather detailed data on the management and outcomes of clinical care in resource-poor settings, and indeed the majority of GNOS participating centres were from highly developed countries, as has been noted with other global collaborative research studies.^22^,^24^ Such studies have previously collected only minimal data on resource availability, instead focusing on the operating theatre setting, an often resource-rich environment. In this study, we have been able to expand on this to understand the entire care pathway of TBI, providing a contextual background to our previously published work.

Various studies have previously examined TBI epidemiology and mortality outcomes across different global regions;^25^,^27^ however, there remains a paucity of data on the availability of resources for TBI care. In Europe, this was in part addressed by the CENTER-TBI study which used a provider profiling questionnaire in an attempt to elucidate these aspects.^28^ However, CENTER-TBI only recruited centres in VH-HDI and H-HDI countries. While high-quality data from LMICs appears to be lacking,^29 30^ there is a clear, growing interest in LMIC trauma care, of which TBI is an important subset.^31 32^ In this study, we report resource data across every HDI strata, demonstrating key findings for the ongoing development of TBI management globally.

There is increasing evidence that trauma is best managed by dedicated trauma systems,^31^ and an estimated >200 000 lives per annum may be saved if they are implemented globally.^33^ While trauma systems have been variably defined,^34^ the interdependence of discrete phases of the care pathway and the influence of inter-phase interactions on overall outcomes have been recognised by an increasing number of international bodies, including the WHO.^5 35 36^ Within our dataset, prehospital care was identified by participating centres overall as the phase of TBI care most in need of improvement, and this view was most pronounced in M-HDI and L-HDI centres. The rationale is clear: improving prehospital TBI care will minimise the extent of secondary brain injury until definitive management can be delivered in hospital.^37^ The vulnerability of the injured brain to even transient episodes of hypoxia and hypotension (and associated secondary brain injury and mortality risk) is well recognised,^38^ yet the majority of L-HDI and M-HDI centres reported that supplemental oxygen and pulse oximetry were mostly unavailable in the prehospital setting. Addressing this critical deficit in acute TBI care may be pivotal in addressing the TBI mortality burden in LMICs, by allowing patients to survive to the hospital for definitive/refined ongoing acute TBI care in theatres/ICU.

Current ICU management of severe TBI benefits from robust guidelines from the Brain Trauma Foundation, which clearly identifies the interventions for which an adequate evidence base currently exists.^38^ Many of these mandate highly specialist equipment requiring potentially expensive per-patient consumables, such as the use of external ventricular drains and/or ICP monitoring. Issues of awareness, training and cost invariably create significant barriers to adherence to these guidelines in LMICs.^39^

Nevertheless, given the complexity of TBI care, pan-pathway improvements are likely to be crucial in delivering a substantial net improvement in TBI outcomes. As an example, it is reasonable to suggest that improving prehospital care alone may improve mortality at the expense of significant morbidity, with profound ethical and societal implications. This is analogous to the effect observed in the RESCUEicp trial of decompressive craniectomy for refractory intracranial hypertension post-TBI, where compared with medical management alone, decompressive craniectomy substantially reduced mortality at 6 and 12 months post-ictus, but with a considerably higher proportion of survivors facing severe disability, that is, being dependent on carers.^40^ Therefore, an isolated improvement in prehospital care might be hypothesised to overwhelm emergency departments with more, and more severely injured patients. This might be expected to divert finite resources away from less severe cases to prioritise intervention in cases which will survive with treatment, but with worse neurological outcomes. An improvement in overall outcomes may only come from concurrent interventions across other phases of TBI care pathways, particularly rehabilitation. This will be essential to mitigate against an increased disability burden associated with acute phase interventions.^31^ Furthermore, it must be considered that the GNOS dataset constitutes facility level data and does not include those patients who died before accessing neurosurgical care. It remains possible that improvements in prehospital care might increase the number of severely injured TBI patients surviving to present to hospital, placing a greater strain on other aspects of the TBI care pathway. Importantly, our figures do not account for mild TBI that does not present to hospital or is not admitted. Mild TBI may often result in only short-term disability at an individual level, but given its far higher incidence, may have a substantial impact on population health and socioeconomic endpoints.

### Strengths and limitations

This study offers comprehensive descriptive global data surrounding resource availability within TBI care systems across a wide geographic and economic spectrum. The completeness of this dataset and the response rate across all HDI country groups makes this an important contribution to existing knowledge of TBI care systems. While the scale and deployment of the study made it impractical to collect meaningful long-term follow-up data, the combination of our primary epidemiological outcome data with the resource stratification described here provides clinicians and policy makers with actionable insights for the improvement of TBI care across a range of settings.

Nevertheless, there are various limitations to consider when interpreting the data. There was inevitable selection bias from the opportunistic recruitment of neurosurgical centres, and as such, it is impossible to have certainty that the centres we recruited were truly representative of the HDI strata to which they belonged. As an example, as centres offering neurosurgical management, respondents were overwhelmingly urban government-funded centres, and as such, practices and resource availability may differ drastically compared with rural or privately funded centres.^38^ The relative parity in operating theatre resources across our cohort is likely to represent an inclusion bias, at least in part, given that all contributing centres were able to provide emergency neurosurgery care (centres not able to provide neurosurgery were excluded). The availability of a minimum package of essential surgery equipment may therefore be considered a surrogate inclusion criterion. As a result, our findings may not be applicable to those centres that admit patients needing TBI management but who transfer them to secondary facilities for neurosurgical intervention when required.^39^

## Conclusion

TBI care is complex and necessarily resource intensive. However, there are substantial global disparities in terms of resource availability within the complex adaptive health systems that cater to TBI, which are likely to explain the divergence in patient outcomes. Improving systems of TBI care requires high-resolution local data, both qualitative and quantitative, in order to design, build and maintain functional care pathways. However, our global snapshot provides compelling evidence that the opinion of neurosurgical providers globally is that improvements outside of the operating room are likely to confer the biggest impact on TBI care. This has profound implications for global and national policymakers and mandates a coordinated approach to global health systems strengthening to improve outcomes after TBI.

## Supplementary material

10.1136/bmjgh-2025-023154 online supplemental file 1

10.1136/bmjgh-2025-023154 online supplemental file 2

## Data Availability

Data are available upon reasonable request.
