# Supplementary material for: Outlining the global variation in resources for traumatic brain injury care: site-level data from the Global Neurotrauma Outcomes Study (GNOS)
Source: BMJ Glob Health. 2026 Apr 28;11(4):e023154. doi: 10.1136/bmjgh-2025-023154 (PMC13141087; doi:10.1136/bmjgh-2025-023154)
Supplement: online supplemental file 1 [file bmjgh-11-4-s001.docx]

### BMJ Global Health Author Reflexivity Statement

Adapted from Morton, B., Vercueil, A., Masekela, R., Heinz, E., Reimer, L., Saleh, S., Kalinga, C., Seekles, M., Biccard, B., Chakaya, J., Abimbola, S., Obasi, A. and Oriyo, N. (2022), Consensus statement on measures to promote equitable authorship in the publication of research from international partnerships. Anaesthesia, 77: 264-276. <https://doi.org/10.1111/anae.15597>

| **Study conceptualisation** | |
| --- | --- |
| 1. How does this study address local research and policy priorities? | This was a global study to describe variation in resources for traumatic brain injury care worldwide, based on input on local needs from a global research partnership of 16 countries. |
| 1. How were local researchers involved in study design? | This was run by a multinational research partnership with HIC/LMIC co-leadership. The international writing group and steering committee were involved in conceptualisation and study design, as well as writing and reviewing the manuscript. |
| **Research management** | |
| 1. How has funding been used to support the local research team(s)? | NIHR funding was used to support local researchers in each partner country of the writing committee, as well as local lead researcher PI time to provide mentorship and support. |
| **Data acquisition and analysis** | |
| 1. How are research staff who conducted data collection acknowledged? | Local research group leads were listed Co-PIs, and a collaborative authorship model for all those submitting clinical data to the study. |
| 1. How have members of the research partnership been provided with access to study data? | All local collaborators retain the rights to their own data. Writing group authors had full access to all the data in the study, if requested, and to all analyses. |
| 1. How were data used to develop analytical skills within the partnership? | Not applicable, as for this study we used descriptive statistics only with which all members were familiar. |
| **Data interpretation** | |
| 1. How have research partners collaborated in interpreting study data? | Multinational writing group and steering committee involved in data analysis. |
| **Drafting and revising for intellectual content** | |
| 1. How were research partners supported to develop writing skills? | Support from other members of the writing group provided as requested by research partners |
| 1. How will research products be shared to address local needs? | The study outlines global resource variation in traumatic brain injury care, providing a platform to support future research, which can be adapted to match local context |
| **Authorship** | |
| 1. How is the leadership, contribution and ownership of this work by LMIC researchers recognised within the authorship? | Multinational writing group and steering committee involved throughout in study write-up and represented in writing group. Collaborative authorship model. |
| 1. How have early career researchers across the partnership been included within the authorship team? | Range of expertise and seniority in the writing group and steering committee. First author is an early career researcher. |
| 1. How has gender balance been addressed within the authorship? | Mix of genders in writing group and in protocol development, represented in the authorship. Collaborative authorship models gender agnostic. Some elements hard to address given the existing gender balance within neurosurgery globally. |
| **Training** | |
| 1. How has the project contributed to training of LMIC researchers? | Support provided in experimental design, analysis and write-up to collaborators across settings |
| **Infrastructure** | |
| 1. How has the project contributed to improvements in local infrastructure? | The project has driven the development of local research teams, and research offices to manage international grants. The findings also support targeted investment to support neurotrauma care. |
| **Governance** | |
| 1. What safeguarding procedures were used to protect local study participants and researchers? | Anonymised study respondents, secure online data capture form, local leadership and governance processes, de-identified results reporting. |
