# Supplementary material for: Outlining the global variation in resources for traumatic brain injury care: site-level data from the Global Neurotrauma Outcomes Study (GNOS)
Source: BMJ Glob Health. 2026 Apr 28;11(4):e023154. doi: 10.1136/bmjgh-2025-023154 (PMC13141087; doi:10.1136/bmjgh-2025-023154)
Supplement: online supplemental file 2 [file bmjgh-11-4-s002.docx]

**Appendix A – site-survey questionnaire**

**Acute Brain Injury Questionnaire**

This survey should be completed by all sites participating in the Global Neurotrauma Outcomes Study. The questions pertain mostly to the resources available locally for the management of acute brain injury. Most of the questions pertain to the management of traumatic brain injury (particularly severe TBI - GCS 3 to 8), however some questions pertain to the management of other forms of acute brain injury such as cerebrovascular disease.

You can print out this document to help you collect the necessary information for this questionnaire. The local study lead should then complete the questionnaire online by following the personal link provided to you when you first registered for the study.

**1. Respondent characteristics**

| What is your name? | … |
| --- | --- |
| Are you medically qualified? | - Yes  - No |
| What is your main specialty? | - Neurosurgery  - Intensive care  - Anaesthesia  - Paediatrics  - Other surgical specialty (e.g. trauma surgery, general surgery)  - Other |
| Do you own a smartphone? | - Yes  - No |
| Does your neurosurgical unit manage just adults, just children or both? | - Adults only  - Children only  - Both |

**2. General**

| Type of healthcare facility | - Government  - Private  - Non-governmental organisation  - Mission  - Industrial |
| --- | --- |
| Do you work in an urban or rural facility? | - Urban  - Rural |
| Type of hospital | - Primary-level hospital  - Secondary-level hospital  - Tertiary-level hospital |
| Do patients have to pay for the care they receive at your institution? | - Yes, all of it  - Yes, some of it  - No, none of it |
| If you had to pick a single phase of TBI management in your institution to improve, which one do you think would have the greatest impact on patient outcome? | - Pre-hospital care  - Initial management in the emergency department  - Surgery  - Anaesthesia  - Intensive care management  - Rehabiltiation |
| Do you use any guidelines to assist with the management of TBI? | - Yes  - No  *If yes, which aspects of the care of TBI patients do you use guidelines for? Tick all that apply.*  🞏 Pre-hospital care  🞏 Emergency department  🞏 Selecting patients for CT head  🞏 Surgery  🞏 Intensive care management  🞏 Treatment of raised intracranial pressure  🞏 Rehabilitation |
| Do you use ICP monitoring for TBI? | - Yes, we feel it is clinically useful in certain patients and always have the resources to do it  - Yes, we feel it is clinically useful in certain patients but only sometimes have the resources to do it  - No, because we never feel it is clinically useful  - No, because we never/very rarely have the resources to do it |
| Do you ever use steroids in the management of TBI? | - Yes  - No |

**3. Initial management – pre-hospital and emergency department**

| Do you have a trauma team who immediately assesses seriously injured patients when they first arrive at your institution? | - All of the time  - Most of the time  - Some of the time  - None of the time | | | |
| --- | --- | --- | --- | --- |
| Do you use the 'WHO Trauma Care checklist' at your institution? | - Yes  - No | | | |
| How often is a pulse oximeter available in the following settings at your institution? | All of the time | Most of the time | Some of the time | None of the time |
| Pre-hospital |  |  |  |  |
| Emergency department |  |  |  |  |
| Operating theatre |  |  |  |  |
| Theatre recovery |  |  |  |  |
| Intensive care |  |  |  |  |
| Ward |  |  |  |  |
| How often is supplemental oxygen available in the following settings at your institution? | All of the time | Most of the time | Some of the time | None of the time |
| Pre-hospital |  |  |  |  |
| Emergency department |  |  |  |  |
| Operating theatre |  |  |  |  |
| Theatre recovery |  |  |  |  |
| Intensive care |  |  |  |  |
| Ward |  |  |  |  |
| Do you have at least one CT scanner in your institution? | - Yes  - No  *Is there always at least one CT scanner functioning at all times at your institution?*  - Yes  - No  *If you answered 'no' (to either of the above), is there a nearby institution which you can always send patients to for emergency CT scans?*  - Yes  - No | | | |

**4. Surgery**

| How many fully trained neurosurgeons are employed by your institution? | … | | | |
| --- | --- | --- | --- | --- |
| How many cranial neurosurgical procedures does your institution perform per year? | … | | | |
| Do you have access to a high speed drill when performing cranial neurosurgical procedures? | - Yes, for all neurosurgical cases  - Yes, for most neurosurgical cases  - Yes, for some neurosurgical cases  - Never | | | |
| How often do you have bone wax available when needed when performing cranial neurosurgical procedures? | - All of the time  - Most of the time  - Some of the time  - None of the time | | | |
| How often do you have intraoperative haemostatic agents available when needed when performing cranial neurosurgical procedures? | - All of the time  - Most of the time  - Some of the time  - None of the time | | | |
| How often do you have access to diathermy when performing cranial neurosurgical procedures? | All of the time | Most of the time | Some of the time | None of the time |
| Monopolar |  |  |  |  |
| Bipolar |  |  |  |  |

**5. Intensive care**

| Does your institution have an intensive care unit? | - Yes  - No  *If no, skip to section 6.* |
| --- | --- |
| Number of intensive care beds available for ADULT neurosurgery patients to be admitted to (if you only treat paediatric patients in your institution, enter 0) | … |
| Number of intensive care beds available for PAEDIATRIC neurosurgery patients to be admitted to (if you only treat adult patients in your institution, enter 0) | … |
| How would you best describe the intensive care unit in your hospital that TBI patients are typically admitted to? | - General ICU  - Neurosurgical ICU  - Neurological/neurosciences ICU  - Trauma ICU  - Neurotrauma ICU  - Surgical ICU  - Medical ICU |
| Do you have a separate paediatric intensive care unit which paediatric TBI patients are managed on? | - Yes  - No, paediatric TBI patients requiring intensive care are managed in the same ICU as adult TBI patients  - We do not manage any paediatric TBI at our institution |
| Do you have access to an ultrasound machine in your intensive care unit? | - Yes  - No |

**6. Severe TBI management**

| How often is a mechanical ventilator available when a SEVERE TBI (GCS 3 to 8) when they need it? | - All of the time  - Most of the time  - Some of the time  - None of the time | | | |
| --- | --- | --- | --- | --- |
| How often are the following monitoring modalities available for SEVERE TBI (GCS 3 to 8) patients when they are needed? | All of the time | Most of the time | Some of the time | None of the time |
| Invasive blood pressure (via arterial line) |  |  |  |  |
| Central venous pressure (via central line) |  |  |  |  |
| End tidal CO_2_ monitoring (via capnography) |  |  |  |  |
| How often are the following treatments available for SEVERE TBI (GCS 3 to 8) patients when they are needed? | All of the time | Most of the time | Some of the time | None of the time |
| Intravenous fluids (crystalloids or colloids) |  |  |  |  |
| Hyperosmolar therapy (e.g. mannitol or hypertonic saline) |  |  |  |  |
| Sedatives |  |  |  |  |
| Muscle relaxants |  |  |  |  |
| Opiates |  |  |  |  |
| Anticonvulsants |  |  |  |  |
| Vasopressors |  |  |  |  |
| Inotropes |  |  |  |  |
| Red blood cell transfusion |  |  |  |  |
| Platelet transfusion |  |  |  |  |
| For patients with SEVERE TBI (GCS 3 to 8), how often are the following tests available when they are needed? | All of the time | Most of the time | Some of the time | None of the time |
| Arterial blood gases |  |  |  |  |
| Electrolytes (including sodium level) |  |  |  |  |
| Full blood count |  |  |  |  |
| Clotting |  |  |  |  |
| Chest radiographs |  |  |  |  |
| How often is enteral (e.g. via NG or PEG) or parenteral feeding to maintain nutritional requirements available for SEVERE TBI (GCS 3 to 8) patients? | - All of the time  - Most of the time  - Some of the time  - None of the time | | | |

**7. Rehabilitation**

| How many of your SEVERE TBI (GCS 3 to 8) patients have access to the following healthcare professionals after the acute period of their illness? | All of the time | Most of the time | Some of the time | None of the time |
| --- | --- | --- | --- | --- |
| Physiotherapist |  |  |  |  |
| Occupational therapist |  |  |  |  |
| Neuropsychologist |  |  |  |  |
| Speech and language therapist |  |  |  |  |
| Dietician |  |  |  |  |
| Rehabilitation medicine physician |  |  |  |  |
| How many of your SEVERE TBI (GCS 3 to 8) patients are followed up in clinic after discharge? | - 0-25%  - 25-50%  - 50-75%  - 75-100% | | | |

**8. Cerebrovascular emergencies**

| Is your institution the unit that manages cerebrovascular emergencies (e.g. subarachnoid haemorrhage) in your region? | - Yes, we manage all cerebrovascular emergencies referred to us  - No, we routinely transfer such cases to a different centre |
| --- | --- |
| Are you able to perform microsurgical clipping of aneurysms at your institution? | - Yes  - No |
| Are you able to coil aneurysms at your institution? | - Yes  - No |
| Are you able to provide thrombectomy for stroke? | - Yes  - No  *If so, do you offer thrombectomy 24 hours a day, 7 days a week?*  - Yes  - No |

**9. Other**

| At your institution, do you maintain a database/registry of traumatic brain injury patients? | - Yes  - No |
| --- | --- |
| Would your institution potentially be interested in participating in an international, longitudinal, hospital-based registry of traumatic brain injury patients? | - Yes  - No |

**1. Respondent characteristics**

**1.1 What is your name?:** Please enter your full name (forename and surname).

**1.2 Are you medically qualified?:** Self-explanatory.

**1.2.1 [if answered ‘Yes’ to 1.2] What is your main specialty?:** If you are a fully trained specialist, please put down your specialty. If you are a resident, please put down the specialty you are training in. If you are a doctor not currently in a training programme, please put down the specialty you are currently working in.

**1.3 Do you own a smartphone?:** Self-explanatory.

**1.4 Does your neurosurgical unit manage just adults, just children or both?:** Self-explanatory.

**2. General**

**2.1 Type of healthcare facility:** Self-explanatory.

**2.2 Do you work in an urban or rural facility?:** What constitutes an ‘urban’ or ‘rural’ environment is left to the discretion of the respondent.

- 1. **Type of hospital:** The definitions of these terms are those used by the WHO in their Disease Control Priorities Project (see <http://www.who.int/management/facility/ReferralDefinitions.pdf> for more detail).
- *Primary-level hospital* - few specialties—mainly internal medicine, obstetrics and gynecology, pediatrics, and general surgery, or just general practice; limited laboratory services available for general but not specialized pathological analysis. Alternative names include district hospital, rural hospital, community hospital, general hospital.
- *Secondary*-*level hospital* - highly differentiated by function with 5 to 10 clinical specialties; size ranges from 200 to 800 beds; often referred to as a provincial hospital. Alternative names include regional hospital, provincial hospital or general hospital.
- *Tertiary-level hospital* - highly specialized staff and technical equipment — for example, cardiology, intensive care unit, and specialized imaging units; clinical services highly differentiated by function; could have teaching activities; size ranges from 300 to 1,500 beds. Alternative names include academic hospital, teaching hospital, university hospital, national hospital or central hospital.

**2.4 Do patients have to pay for the care they receive at your facility?:** Self-explanatory.

**2.5 If you had to pick a single phase of TBI management in your institution to improve, which one do you think would have the greatest impact on patient outcome?:** Self explanatory.

**2.6 Do you use any guidelines to assist with the management of TBI?:** Self-explanatory.

**2.6.1 If yes, which aspects of the care of TBI patients do you use guidelines for? Tick all that apply.** You can tick more than one option. Note that although you might have only one guideline at your institution (for example, a guideline for the management of TBI in ICU), it may cover multiple topics in this question (for example, if the above example of a guideline for TBI in ICU contained guidance on the treatment of raised ICP as well as other ICU measures such as preventing pressure sores, then it would be appropriate to tick both ‘Intensive care management’ and ‘ICP management’).

**2.7 Do you ever use steroids in the management of TBI?:** In our practice, we do not ever use steroids in the management of acute TBI as the results of the CRASH trial suggest this increases mortality significantly. However, we are aware certain units throughout the world continue to do this for particular indications and we are interested to see how prevalent this practice is. Your answers to this question, as to all others in this survey, will remain anonymous. Note that this question does NOT apply to patients with chronic subdural haematomas only - it only applies to truly acute cases of TBI (e.g. extradural haematoma, subdural haematoma, diffuse axonal injury, contusions).

**2.8 Do you use ICP monitoring for TBI?:** Self-explanatory.

**3. Initial management – pre-hospital and emergency department**

**3.1 Do you have a trauma team who immediately assesses seriously injured patients when they first arrive at your institution?:** In many hospitals around the world, when a seriously injured patient first arrives at their hospital they will be immediately assessed by a 'trauma team' - a multi-disciplinary group of healthcare workers who collectively work together on the assessment and treatment of severely injured patients. The aims of the trauma team are to resuscitate and thereby stabilise the patient while ascertaining the nature and extent of their injuries and preparing the patient for transfer to a place where definitive care can be delivered (such as intensive care, theatre, the ward or another hospital). There is considerable variation worldwide in who makes up a trauma team. The group may consist of some or all of an emergency department physician, general surgeon, orthopaedic surgeon, anaesthetist, anaesthetic assistant and nurses.

**3.2** **Do you use the ‘WHO Trauma Care checklist’ at your institution?:** The WHO Trauma Care checklist is a tool designed to improve outcomes following major trauma throughout the world, initially released in 2016. It has been validated in a wide variety of settings. It is used in the initial stages of the assessment and resuscitation of a seriously injured patient, typically in the emergency department or its equivalent. If the questionnaire is being answered by a surgeon or anaesthetist, it may be necessary to consult with the doctors (or other healthcare professionals, as appropriate) staffing the emergency department at your institution. More information and a copy of the checklist can be found at <http://www.who.int/emergencycare/trauma-care-checklist-launch/en/>.

**3.3 How often is a pulse oximeter available in the following settings at your institution?:** Self-explanatory.

**3.4** **How often is supplemental oxygen available in the following settings at your institution?:** Self-explanatory.

**3.5 Do you have at least one CT scanner at your institution?:** Self-explanatory.

**3.5.1 [If answered ‘No’ to 3.3] Is there always at least one CT scanner functioning at all times at your institution?:** Some institutions throughout the world may have one or more CT scanners on site, but these may not be available at all times due to equipment malfunction.

**3.5.2 [If answered ‘No’ to 3.3 or 3.4] If you answered 'no', is there a nearby institution which you can always send patients to for emergency CT scans?:** In some institutions worldwide, although they may not always have a CT scanner functioning at all times at their own institution, there are hospitals/clinics nearby in the same town/city/region where they can usually send patients to obtain the appropriate imaging on an emergency basis. Please only answer ‘yes’ to this question if you are able to obtain URGENT CT scans from these nearby institutions (i.e. within a few hours) and this option is available the majority of the time.

**4. Surgery**

**4.1 How many fully trained neurosurgeons are employed by your institution?:** Self-explanatory. This refers to individuals who have completed a neurosurgical training programme.

**4.2 How many cranial neurosurgical procedures does your institution perform per year?:** Where an official annual figure for the past year exists, please provide this. Otherwise, please estimate by determining the total number of ALL cranial neurosurgical procedures (for TBI AND any other cranial pathology) performed during your site's 1 month study inclusion period and multiplying by 12.

**4.3 Do you have access to a high speed drill when performing cranial neurosurgical procedures?:** Self-explanatory.

**4.4 How often do you have bone wax available when needed when performing cranial neurosurgical procedures?:** Self-explanatory.

**4.5 How often do you have intraoperative haemostatic agents available when needed when performing cranial neurosurgical procedures?:** Examples of common agents used in neurosurgery include Surgicel, absorbable gelatin sponges (e.g. GELFOAM or SPONGISTAN) and fibrin sealants.

**4.6 How often do you have access to diathermy when performing cranial neurosurgical procedures?:** Self-explanatory.

**5. Intensive care**

**5.1 Does your institution have an intensive care unit?:** Self-explanatory. We accept that there is considerable variation in what is defined as an intensive care unit (ICU) worldwide, particularly in low resource settings. For the purposes of this survey, we have chosen to use the definition of an ‘intensive care unit’ used by Haniffa et al. (1): units that have at least 1 ventilator and where patients are expected to be admitted for at least 24 hours. Note that ad hoc “high dependency” areas in a ward with some extra monitoring, postoperative “recovery” areas where a patient may be ventilated for a few hours and emergency treatment units similar to resuscitation areas in accident and emergency departments in high-income countries (where treatment is limited to initial stabilization before transfer to a definitive critical care area) are all excluded from this definition.

**5.2 Number of intensive care beds available for ADULT neurosurgery patients to be admitted to (if you only treat paediatric patients in your institution, enter 0):** Self-explanatory.

**5.3 Number of intensive care beds available for PAEDIATRIC neurosurgery patients to be admitted to (if you only treat adult patients in your institution, enter 0):** Self-explanatory.

**5.4 How would you best describe the intensive care unit in your hospital that TBI patients are typically admitted to?:** If TBI patients are typically admitted to more than 1 of the ICUs listed below, please tick the one that the majority of TBI patients are admitted to. If you feel that the ICU fits more than one of the descriptions (for example, an ICU that deals with major trauma and acute care surgery could either fit into the ‘trauma ICU’ or ‘surgical ICU’ designations) then please select the one that best fits.

- *General ICU* – select this option if you only have a single intensive care unit in your hospital.
- *Neurosurgical ICU* – for patients admitted with neurosurgical pathology and/or following neurosurgical intervention. Typical pathology includes TBI, subarachnoid haemorrhage (SAH), postoperative craniotomies for tumours and/or major spine surgery. Typically staffed by neurosurgeons, neurointensivists and/or anaesthetists.
- *Neurosciences/neurological ICU* – for patients admitted with neurosurgical, neurological and/or stroke related pathology. Typical pathology includes TBI, SAH, ischaemic stroke, spinal cord injury, meningitis and/or encephalitis. Typically staffed by neurosurgeons, neurologists, neurointensivists and/or anaesthetists.
- *Trauma ICU* – for patients admitted following major trauma. Typically staffed by trauma surgeons, general surgeons, intensivists and/or anaesthetists.
- *Neurotrauma ICU* – for patients admitted following traumatic brain injury (and, in some units, spinal cord injury as well). Typically staffed by neurosurgeons, trauma surgeons, general surgeons, intensivists and/or anaesthetists.
- *Surgical ICU* – for patients admitted following major surgery or with surgical pathology being managed conservatively (including potentially traumatic injuries). Typically staffed by general surgeons, trauma surgeons, intensivists and anaesthetists.
- *Medical ICU* – for patients admitted with serious medical conditions. Typically staffed by physicians, intensivists and/or anaesthetists.

**5.5 Do you have a separate paediatric intensive care unit which paediatric TBI patients are managed on?:** Self-explanatory.

**5.4 Do you have access to an ultrasound machine in your intensive care unit?** Self-explanatory. Note that if you do not have an ultrasound machine specifically in the intensive care unit but there is an ultrasound machine in a nearby area (e.g. emergency department or operating theatres) that you can borrow when you need it the majority of the time, then you can also answer ‘yes’ to this question.

**6. Severe TBI management**

Most of the following questions pertain to the availability of resources for the management of severe TBI patients in your institution. We are interested in what resources are actually able to be used for the care of TBI patients - for example, if your hospital owns a piece of equipment but it never gets used then this would not be considered available.

We accept that in hospitals which treat both adult and paediatric patients that resources for intensive care may differ significantly between these 2 groups. As such, if your institution treats BOTH ADULT AND PAEDIATRIC PATIENTS then, unless the question specifically asks about paediatric management, please simply answer the following questions on severe TBI management as they apply to your management of ADULT patients ONLY.

If your institution treats ADULT patients only, please simply answer the following questions on severe TBI management as they apply to your management of ADULT patients. If your institution treats PAEDIATRIC patients only, please simply answer the following questions on severe TBI management as they apply to your management of PAEDIATRIC patients.

**6.1 How often is a mechanical ventilator available when a SEVERE TBI (GCS 3 to 8) patient needs it?:** Self-explanatory.

**6.2 How often are the following monitoring modalities available for SEVERE TBI patients when they are needed?:** Self-explanatory.

**6.3 How often are the following treatments available for SEVERE TBI (GCS 3 to 8) patients when they are needed?:** Self-explanatory. Note that if you routinely transufse ‘whole blood’ in place of ‘red blood cells’, please answer this question as if ‘red blood cells’ was instead replaced by ‘whole blood’.

**6.4 For patients with SEVERE TBI (GCS 3 to 8), how often are the following tests available when they are needed?:** Self-explanatory. Note that ‘full blood count’ is also known as ‘complete blood count’ or ‘whole blood count’.

**6.5 How often is enteral (e.g. via NG or PEG) or parenteral feeding to maintain requirements for protein and calories available for SEVERE TBI (GCS 3 to 8) patients?:** Self-explanatory.

**7. Rehabilitation**

**7.1 How many of your SEVERE TBI (GCS 3 to 8) patients have access to the following healthcare professionals after the acute period of their illness?:**

- *Physiotherapist* – the WHO defines a physiotherapist as a healthcare worker who is able to ‘assess, plan and implement rehabilitative programs that improve or restore human motor functions, maximize movement ability, relieve pain syndromes, and treat or prevent physical challenges associated with injuries, diseases and other impairments. They apply a broad range of physical therapies and techniques such as movement, ultrasound, heating, laser and other techniques. They may develop and implement programmes for screening and prevention of common physical ailments and disorders.’
- *Occupational therapist* – the World Federation of Occupational Therapists defines occupational therapy as ‘a client-centred health profession concerned with promoting health and well being through occupation. The primary goal of occupational therapy is to enable people to participate in the activities of everyday life. Occupational therapists achieve this outcome by working with people and communities to enhance their ability to engage in the occupations they want to, need to, or are expected to do, or by modifying the occupation or the environment to better support their occupational engagement
- *Neuropsychologist* – the National Academy of Neuropsychology defines a neuropsychologist as ‘a professional within the field of psychology with special expertise in the applied science of brain-behavior relationships. Clinical neuropsychologists use this knowledge in the assessment, diagnosis, treatment, and/or rehabilitation of patients across the lifespan with neurological, medical, neurodevelopmental and psychiatric conditions, as well as other cognitive and learning disorders. The clinical neuropsychologist uses psychological, neurological, cognitive, behavioral, and physiological principles, techniques and tests to evaluate patients’ neurocognitive, behavioral, and emotional strengths and weaknesses and their relationship to normal and abnormal central nervous system functioning. The clinical neuropsychologist uses this information and information provided by other medical/healthcare providers to identify and diagnose neurobehavioral disorders, and plan and implement intervention strategies.’
- *Speech and language therapist* – the Royal College of Speech and Language Therapists define Speech and Language Therapy as ‘concerned with the management of disorders of speech, language, communication and swallowing in children and adults.’
- *Dietician* – the WHO defines a dietician as a healthcare worker who is able to ‘assess, plan and implement programs to enhance the impact of food and nutrition on human health. They may conduct research, assessments and education to improve nutritional levels among individuals and communities.’
- *Rehabilitation medicine physician* – ‘rehabilitation medicine’ is a specialty of medicine (therefore requires a medical degree and undertaking a postgraduate training programme in this specialty) and is also referred to as physical medicine or physiatry.

**7.2 How many of your SEVERE TBI (GCS 3 to 8) patients are followed up in clinic after discharge?:** This refers to ANY follow up whatsoever at all after hospital discharge.

**8. Cerebrovascular emergencies**

The following questions are on the surgical and endovascular management of cerebrovascular emergencies. We are interested to see if there are significant differences in the resources available for the management of TBI and other forms of acute brain injury which require immediate treatment (such as cerebrovascular pathology) in the same institution.

**8.1 Is your institution a unit that manages cerebrovascular emergencies (e.g. subarachnoid haemorrhage) in your region?:** In some regions, your institution may receive patients who have had cerebrovascular emergency pathologies such as subarachnoid haemorrhage and immediately refer them to a nearby specialist unit that is equipped to provide definitive management (such as coiling or clipping) – in this case, you should answer ‘no’ to this question. However, if you are in a country or region (particularly in low resource settings) where your institution is the main or one of the main neurosurgical units, often receives patients with cerebrovascular emergencies, is unable to treat such emergencies but there is no nearby unit that is able to provide definitive management, then you should still answer ‘yes’ to this question.

**8.2 Are you able to perform microsurgical clipping of aneurysms at your institution?:** You should only answer ‘Yes’ if you are regularly performing this procedure at your institution. For example, if a cerebrovascular neurosurgeon visits from another country for a short term visit annually to clip aneurysms then you should still answer ‘No’.

**8.3 Are you able to coil aneurysms at your institution?:** As per question 7.2, you should only answer ‘yes’ to this question if you are regularly performing this procedure at your institution.

**8.4 Are you able to provide thrombectomy for stroke?:** Self-explanatory. If you are regularly performing this procedure, you should answer ‘Yes’ to this question. You should still answer ‘Yes’ even if you do not currently offer this service 24 hours a day, 7 days a week – this will be clarified in question

**8.4.1 If so, do you offer thrombectomy 24 hours a day, 7 days a week?:** Self-explanatory.

**9. Other**

**9.1 At your institution, do you maintain a database/registry of traumatic brain injury patients?:** Self-explanatory.

**9.2** **Would your institution potentially be interested in participating in an international, longitudinal, hospital-based registry of traumatic brain injury patients?:** Self-explanatory. Answering this question ‘Yes’ indicates only that you may be interested in participating – it does not mean you have to participate.

**References**

1. Haniffa R, De Silva AP, Iddagoda S, Batawalage H, De Silva ST, Mahipala PG, et al. A cross-sectional survey of critical care services in Sri Lanka: a lower middle-income country. J Crit Care. 2014;29(5):764-8.

**________________________________________________________________**

**Appendix A| Full site-survey questionnaire for GNOS.** The full 50-point questionnaire, delivered electronically via Qualtrics is reproduced here.

**Appendix B – remaining site-survey data**

|  | | VH-HDI (N=78) | | H-HDI (N=33) | | M-HDI (N=28) | | L-HDI (N=14) | | Total (N=153) | p value |
| --- | --- | --- | --- | --- | --- | --- | --- | --- | --- | --- | --- |
| INITIAL MANAGEMENT – PRE-HOSPITAL AND EMERGENCY DEPARTMENT | | | | | | | | | | | |
| Do you have a trauma team who immediately assesses seriously injured patients when they first arrive at your trauma institution? |  | | |  | |  | |  | |  | < 0.001 |
| All of the time | | 65 (83%) | | 17 (52%) | | 17 (61%) | | 4 (29%) | | 103 (67%) |  |
| Most of the time | | 4 (5%) | | 9 (27%) | | 9 (32%) | | 7 (50%) | | 29 (19%) |  |
| Some of the time | | 1 (1%) | | 3 (9%) | | 1 (4%) | | 2 (14%) | | 7 (5%) |  |
| None of the time | | 8 (10%) | | 4 (12%) | | 1 (4%) | | 1 (7%) | | 14 (9%) |  |
|  | |  | |  | |  | |  | |  |  |
| Do you use the 'WHO Trauma Care checklist' at your institution? | | 27 (35%) | | 3 (9%) | | 14 (50%) | | 4 (29%) | | 48 (31%) | 0.006 |
|  | |  | |  | |  | |  | |  |  |
| How often is a pulse oximeter available in the following locations in your institution? | | |  | |  | |  | |  |  |  |
| Pre-hospital | |  | |  | |  | |  | |  | < 0.001 |
| All of the time | | 69 (88%) | | 12 (36%) | | 3 (11%) | | 0 (0%) | | 84 (55%) |  |
| Most of the time | | 8 (10%) | | 8 (24%) | | 5 (18%) | | 0 (0%) | | 21 (14%) |  |
| Some of the time | | 1 (1%) | | 13 (39%) | | 13 (46%) | | 5 (36%) | | 32 (21%) |  |
| None of the time | | 0 (0%) | | 0 (0%) | | 7 (25%) | | 9 (64%) | | 16 (10%) |  |
| Emergency department | |  | |  | |  | |  | |  | < 0.001 |
| All of the time | | 78 (100%) | | 26 (79%) | | 19 (68%) | | 6 (43%) | | 129 (84%) |  |
| Most of the time | | 0 (0%) | | 7 (21%) | | 7 (25%) | | 4 (29%) | | 18 (12%) |  |
| Some of the time | | 0 (0%) | | 0 (0%) | | 2 (7%) | | 4 (29%) | | 6 (4%) |  |
| None of the time | | 0 (0%) | | 0 (0%) | | 0 (0%) | | 0 (0%) | | 0 (0%) |  |
| Operating theatre | |  | |  | |  | |  | |  | < 0.001 |
| All of the time | | 78 (100%) | | 33 (100%) | | 28 (100%) | | 12 (86%) | | 151 (99%) |  |
| Most of the time | | 0 (0%) | | 0 (0%) | | 0 (0%) | | 2 (14%) | | 2 (1%) |  |
| Some of the time | | 0 (0%) | | 0 (0%) | | 0 (0%) | | 0 (0%) | | 0 (0%) |  |
| None of the time | | 0 (0%) | | 0 (0%) | | 0 (0%) | | 0 (0%) | | 0 (0%) |  |
| Recovery | |  | |  | |  | |  | |  | < 0.001 |
| All of the time | | 78 (100%) | | 29 (88%) | | 25 (89%) | | 9 (64%) | | 141 (92%) |  |
| Most of the time | | 0 (0%) | | 4 (12%) | | 2 (7%) | | 4 (29%) | | 10 (7%) |  |
| Some of the time | | 0 (0%) | | 0 (0%) | | 1 (4%) | | 1 (7%) | | 2 (1%) |  |
| None of the time | | 0 (0%) | | 0 (0%) | | 0 (0%) | | 0 (0%) | | 0 (0%) |  |
| ICU | |  | |  | |  | |  | |  | 0.001 |
| All of the time | | 78 (100%) | | 32 (97%) | | 25 (89%) | | 11 (79%) | | 146 (95%) |  |
| Most of the time | | 0 (0%) | | 1 (3%) | | 3 (11%) | | 3 (21%) | | 7 (5%) |  |
| Some of the time | | 0 (0%) | | 0 (0%) | | 0 (0%) | | 0 (0%) | | 0 (0%) |  |
| None of the time | | 0 (0%) | | 0 (0%) | | 0 (0%) | | 0 (0%) | | 0 (0%) |  |
| Ward | |  | |  | |  | |  | |  | < 0.001 |
| All of the time | | 59 (76%) | | 16 (48%) | | 11 (39%) | | 4 (29%) | | 90 (59%) |  |
| Most of the time | | 11 (14%) | | 4 (12%) | | 8 (29%) | | 2 (14%) | | 25 (16%) |  |
| Some of the time | | 7 (9%) | | 10 (30%) | | 9 (32%) | | 8 (57%) | | 34 (22%) |  |
| None of the time | | 1 (1%) | | 3 (9%) | | 0 (0%) | | 0 (0%) | | 4 (3%) |  |
|  | |  | |  | |  | |  | |  |  |
| How often is supplemental oxygen available in the following settings at your institution? | |  | |  | |  | |  | |  |  |
| Pre-hospital | |  | |  | |  | |  | |  | < 0.001 |
| All of the time | | 69 (88%) | | 14 (42%) | | 7 (25%) | | 0 (0%) | | 90 (59%) |  |
| Most of the time | | 8 (10%) | | 13 (39%) | | 7 (25%) | | 1 (7%) | | 29 (19%) |  |
| Some of the time | | 1 (1%) | | 6 (18%) | | 11 (39%) | | 6 (43%) | | 24 (16%) |  |
| None of the time | | 0 (0%) | | 0 (0%) | | 3 (11%) | | 7 (50%) | | 10 (7%) |  |
| Emergency department | |  | |  | |  | |  | |  | < 0.001 |
| All of the time | | 77 (99%) | | 33 (100%) | | 26 (93%) | | 8 (57%) | | 144 (94%) |  |
| Most of the time | | 1 (1%) | | 0 (0%) | | 1 (4%) | | 5 (36%) | | 7 (5%) |  |
| Some of the time | | 0 (0%) | | 0 (0%) | | 1 (4%) | | 1 (7%) | | 2 (1%) |  |
| None of the time | | 0 (0%) | | 0 (0%) | | 0 (0%) | | 0 (0%) | | 0 (0%) |  |
| Operating theatre | |  | |  | |  | |  | |  |  |
| All of the time | | 78 (100%) | | 33 (100%) | | 28 (100%) | | 12 (86%) | | 151 (99%) | < 0.001 |
| Most of the time | | 0 (0%) | | 0 (0%) | | 0 (0%) | | 2 (14%) | | 2 (1%) |  |
| Some of the time | | 0 (0%) | | 0 (0%) | | 0 (0%) | | 0 (0%) | | 0 (0%) |  |
| None of the time | | 0 (0%) | | 0 (0%) | | 0 (0%) | | 0 (0%) | | 0 (0%) |  |
| Recovery | |  | |  | |  | |  | |  | < 0.001 |
| All of the time | | 78 (100%) | | 32 (97%) | | 28 (100%) | | 10 (71%) | | 148 (97%) |  |
| Most of the time | | 0 (0%) | | 1 (3%) | | 0 (0%) | | 3 (21%) | | 4 (3%) |  |
| Some of the time | | 0 (0%) | | 0 (0%) | | 0 (0%) | | 1 (7%) | | 1 (1%) |  |
| None of the time | | 0 (0%) | | 0 (0%) | | 0 (0%) | | 0 (0%) | | 0 (0%) |  |
| ICU | |  | |  | |  | |  | |  | < 0.001 |
| All of the time | | 78 (100%) | | 33 (100%) | | 28 (100%) | | 12 (86%) | | 151 (99%) |  |
| Most of the time | | 0 (0%) | | 0 (0%) | | 0 (0%) | | 2 (14%) | | 2 (1%) |  |
| Some of the time | | 0 (0%) | | 0 (0%) | | 0 (0%) | | 0 (0%) | | 0 (0%) |  |
| None of the time | | 0 (0%) | | 0 (0%) | | 0 (0%) | | 0 (0%) | | 0 (0%) |  |
| Ward | |  | |  | |  | |  | |  | < 0.001 |
| All of the time | | 71 (91%) | | 22 (67%) | | 22 (79%) | | 5 (36%) | | 120 (78%) |  |
| Most of the time | | 6 (8%) | | 5 (15%) | | 5 (18%) | | 6 (43%) | | 22 (14%) |  |
| Some of the time | | 1 (1%) | | 5 (15%) | | 1 (4%) | | 2 (14%) | | 9 (6%) |  |
| None of the time | | 0 (0%) | | 1 (3%) | | 0 (0%) | | 1 (7%) | | 2 (1%) |  |
|  | |  | |  | |  | |  | |  |  |
| SURGERY | |  | |  | |  | |  | |  |  |
|  | |  | |  | |  | |  | |  |  |
| Access to neurosurgical equipment | |  | |  | |  | |  | |  |  |
| Diathermy - monopolar | |  | |  | |  | |  | |  | 0.009 |
| All of the time | | 75 (96%) | | 28 (85%) | | 28 (100%) | | 10 (71%) | | 141 (92%) |  |
| Most of the time | | 3 (4%) | | 3 (9%) | | 0 (0%) | | 4 (29%) | | 10 (7%) |  |
| Some of the time | | 0 (0%) | | 1 (3%) | | 0 (0%) | | 0 (0%) | | 1 (0.5%) |  |
| None of the time | | 0 (0%) | | 1 (3%) | | 0 (0%) | | 0 (0%) | | 1 (0.5%) |  |
| Diathermy - bipolar | |  | |  | |  | |  | |  | < 0.001 |
| All of the time | | 77 (99%) | | 30 (91%) | | 27 (96%) | | 7 (50%) | | 141 (92%) |  |
| Most of the time | | 0 (0%) | | 1 (3%) | | 1 (4%) | | 6 (43%) | | 8 (5%) |  |
| Some of the time | | 1 (1%) | | 1 (3%) | | 0 (0%) | | 0 (0%) | | 2 (1%) |  |
| None of the time | | 0 (0%) | | 1 (3%) | | 0 (0%) | | 1 (7%) | | 2 (1%) |  |
|  | |  | |  | |  | |  | |  |  |
| INTENSIVE CARE | |  | |  | |  | |  | |  |  |
|  | |  | |  | |  | |  | |  |  |
| Do you have a separate paediatric intensive care unit which paediatric TBI patients are managed on? | |  | |  | |  | |  | |  | < 0.001 |
| Yes | | 43 (55%) | | 13 (39%) | | 8 (29%) | | 4 (29%) | | 68 (44%) |  |
| No, paediatric TBI patients requiring intensive care are typically managed in the same ICU as adult TBI patients | | 13 (17%) | | 12 (36%) | | 19 (68%) | | 9 (64%) | | 53 (35%) |  |
| We do not manage any paediatric TBI at our institution | | 22 (28%) | | 8 (24%) | | 1 (4%) | | 1 (7%) | | 32 (21%) |  |
|  | |  | |  | |  | |  | |  |  |
| Access to an ultrasound machine in ICU | | 73 (94%) | | 25 (76%) | | 17 (61%) | | 10 (71%) | | 125 (82%) | < 0.001 |
|  | |  | |  | |  | |  | |  |  |
| Do you ever use steroids in the management of TBI? | | 15 (19%) | | 6 (18%) | | 6 (21%) | | 2 (14%) | | 29 (19%) | 0.955 |
|  | |  | |  | |  | |  | |  |  |
| MANAGING SEVERE TBI (GCS 3-8) | |  | |  | |  | |  | |  |  |
|  | |  | |  | |  | |  | |  |  |
| Availability of treatments for severe TBI when needed | |  | |  | |  | |  | |  |  |
| Intravenous fluids | |  | |  | |  | |  | |  | 0.006 |
| All of the time | | 77 (99%) | | 33 (100%) | | 28 (100%) | | 12 (86%) | | 150 (98%) |  |
| Most of the time | | 1 (1%) | | 0 (0%) | | 0 (0%) | | 2 (14%) | | 3 (2%) |  |
| Some of the time | | 0 (0%) | | 0 (0%) | | 0 (0%) | | 0 (0%) | | 0 (0%) |  |
| None of the time | | 0 (0%) | | 0 (0%) | | 0 (0%) | | 0 (0%) | | 0 (0%) |  |
| Opiates | |  | |  | |  | |  | |  | < 0.001 |
| All of the time | | 77 (99%) | | 30 (91%) | | 24 (86%) | | 8 (57%) | | 139 (91%) |  |
| Most of the time | | 1 (1%) | | 2 (6%) | | 1 (4%) | | 4 (29%) | | 8 (5%) |  |
| Some of the time | | 0 (0%) | | 0 (0%) | | 3 (11%) | | 2 (14%) | | 5 (3%) |  |
| None of the time | | 0 (0%) | | 1 (3%) | | 0 (0%) | | 0 (0%) | | 1 (1%) |  |
| Anticonvulsants | |  | |  | |  | |  | |  | < 0.001 |
| All of the time | | 77 (99%) | | 29 (88%) | | 27 (96%) | | 8 (57%) | | 141 (92%) |  |
| Most of the time | | 0 (0%) | | 3 (9%) | | 0 (0%) | | 6 (43%) | | 9 (6%) |  |
| Some of the time | | 1 (1%) | | 1 (3%) | | 1 (4%) | | 0 (0%) | | 3 (2%) |  |
| None of the time | | 0 (0%) | | 0 (0%) | | 0 (0%) | | 0 (0%) | | 0 (0%) |  |
| Vasopressors | |  | |  | |  | |  | |  | < 0.001 |
| All of the time | | 77 (99%) | | 29 (88%) | | 23 (82%) | | 6 (43%) | | 135 (88%) |  |
| Most of the time | | 1 (1%) | | 2 (6%) | | 3 (11%) | | 5 (36%) | | 11 (7%) |  |
| Some of the time | | 0 (0%) | | 2 (6%) | | 2 (7%) | | 3 (21%) | | 7 (5%) |  |
| None of the time | | 0 (0%) | | 0 (0%) | | 0 (0%) | | 0 (0%) | | 0 (0%) |  |
| Inotropes | |  | |  | |  | |  | |  | < 0.001 |
| All of the time | | 77 (99%) | | 29 (88%) | | 24 (86%) | | 5 (36%) | | 135 (88%) |  |
| Most of the time | | 1 (1%) | | 2 (6%) | | 2 (7%) | | 6 (43%) | | 11 (7%) |  |
| Some of the time | | 0 (0%) | | 2 (6%) | | 2 (7%) | | 3 (21%) | | 7 (5%) |  |
| None of the time | | 0 (0%) | | 0 (0%) | | 0 (0%) | | 0 (0%) | | 0 (0%) |  |
| Platelet transfusion | |  | |  | |  | |  | |  | < 0.001 |
| All of the time | | 71 (91%) | | 22 (67%) | | 20 (71%) | | 3 (21%) | | 116 (76%) |  |
| Most of the time | | 4 (5%) | | 8 (24%) | | 6 (21%) | | 3 (21%) | | 21 (14%) |  |
| Some of the time | | 3 (4%) | | 3 (9%) | | 2 (7%) | | 4 (29%) | | 12 (8%) |  |
| None of the time | | 0 (0%) | | 0 (0%) | | 0 (0%) | | 4 (29%) | | 4 (3%) |  |
|  | |  | |  | |  | |  | |  |  |
| Availability of investigations for severe TBI when needed | |  | |  | |  | |  | |  |  |

| Central venous pressure (via central venous catheter) |  |  |  |  |  | < 0.001 |
| --- | --- | --- | --- | --- | --- | --- |
| All the time | 75 (96%) | 20 (61%) | 14 (50%) | 1 (7%) | 110 (72%) |  |
| Most of the time | 2 (3%) | 6 (18%) | 6 (21%) | 1 (7%) | 15 (10%) |  |
| Some of the time | 1 (1%) | 5 (15%) | 5 (18%) | 5 (36%) | 16 (10%) |  |
| None of the time | 0 (0%) | 2 (6%) | 3 (11%) | 7 (50%) | 12 (8%) |  |

| Full blood count |  |  |  |  |  | < 0.001 |
| --- | --- | --- | --- | --- | --- | --- |
| All of the time | 77 (99%) | 32 (97%) | 27 (96%) | 9 (64%) | 145 (95%) |  |
| Most of the time | 1 (1%) | 1 (3%) | 1 (4%) | 4 (29%) | 7 (4%) |  |
| Some of the time | 0 (0%) | 0 (0%) | 0 (0%) | 1 (7%) | 1 (1%) |  |
| None of the time | 0 (0%) | 0 (0%) | 0 (0%) | 0 (0%) | 0 (0%) |  |
| Clotting |  |  |  |  |  | < 0.001 |
| All of the time | 77 (99%) | 30 (91%) | 24 (86%) | 6 (43%) | 137 (90%) |  |
| Most of the time | 1 (1%) | 2 (6%) | 4 (14%) | 5 (36%) | 12 (8%) |  |
| Some of the time | 0 (0%) | 1 (3%) | 0 (0%) | 3 (21%) | 4 (2%) |  |
| None of the time | 0 (0%) | 0 (0%) | 0 (0%) | 0 (0%) | 0 (0%) |  |
| Blood glucose |  |  |  |  |  | < 0.001 |
| All of the time | 78 (100%) | 32 (97%) | 27 (96%) | 10 (71%) | 147 (96%) |  |
| Most of the time | 0 (0%) | 1 (3%) | 0 (0%) | 2 (14%) | 3 (2%) |  |
| Some of the time | 0 (0%) | 0 (0%) | 1 (4%) | 2 (14%) | 3 (2%) |  |
| None of the time | 0 (0%) | 0 (0%) | 0 (0%) | 0 (0%) | 0 (0%) |  |
| Chest radiograph |  |  |  |  |  | 0.011 |
| All of the time | 75 (96%) | 29 (88%) | 23 (82%) | 9 (64%) | 136 (89%) |  |
| Most of the time | 3 (4%) | 3 (9%) | 4 (14%) | 3 (21%) | 13 (8%) |  |
| Some of the time | 0 (0%) | 1 (3%) | 1 (4%) | 1 (7%) | 3 (2%) |  |
| None of the time | 0 (0%) | 0 (0%) | 0 (0%) | 1 (7%) | 1 (1%) |  |
|  |  |  |  |  |  |  |
| REHABILITATION |  |  |  |  |  |  |
| How many of your severe TBI patients are followed up in clinic after discharge? |  |  |  |  |  | 0.004 |
| 0-25% | 13 (17%) | 8 (24%) | 1 (4%) | 3 (21%) | 25 (16%) |  |
| 25-50% | 9 (12%) | 4 (12%) | 5 (18%) | 6 (43%) | 24 (16%) |  |
| 50-75% | 11 (14%) | 9 (27%) | 11 (39%) | 2 (14%) | 33 (22%) |  |
| 75-100% | 45 (58%) | 12 (36%) | 11 (39%) | 3 (21%) | 71 (46%) |  |

**Appendix B| Remainder of qualitative data collected for GNOS.** This covers each of the phases of care described in the main paper. Data are stratified by HDI tier of the countries in which each participating centre was situated in. Data are presented as n (%), and p values are derived from χ² analyses.
